# Optimizing ChatGPT’s Interpretation and Reporting of Delirium Assessment Outcomes: Exploratory Study

**DOI:** 10.2196/51383

**Published:** 2024-10-01

**Authors:** Yong K Choi, Shih-Yin Lin, Donna Marie Fick, Richard W Shulman, Sangil Lee, Priyanka Shrestha, Kate Santoso

**Affiliations:** 1 Department of Health Information Management School of Health and Rehabilitation Sciences University of Pittsburgh Pittsburgh, PA United States; 2 Rory Meyers College of Nursing New York University New York, NY United States; 3 Ross and Carol Nese College of Nursing Penn State University University Park, PA United States; 4 Trillium Health Partners Toronto, ON Canada; 5 Division of Geriatric Psychiatry, Department of Psychiatry Faculty of Medicine University of Toronto Toronto, ON Canada; 6 Department of Emergency Medicine University of Iowa Carver College of Medicine Iowa City, IA United States; 7 Community of Policy Populations and Systems George Washington University School of Nursing Washington, DC United States

**Keywords:** generative artificial intelligence, generative AI, large language models, ChatGPT, delirium detection, Sour Seven Questionnaire, prompt engineering, clinical vignettes, medical education, caregiver education

## Abstract

**Background:**

Generative artificial intelligence (AI) and large language models, such as OpenAI’s ChatGPT, have shown promising potential in supporting medical education and clinical decision-making, given their vast knowledge base and natural language processing capabilities. As a general purpose AI system, ChatGPT can complete a wide range of tasks, including differential diagnosis without additional training. However, the specific application of ChatGPT in learning and applying a series of specialized, context-specific tasks mimicking the workflow of a human assessor, such as administering a standardized assessment questionnaire, followed by inputting assessment results in a standardized form, and interpretating assessment results strictly following credible, published scoring criteria, have not been thoroughly studied.

**Objective:**

This exploratory study aims to evaluate and optimize ChatGPT’s capabilities in administering and interpreting the Sour Seven Questionnaire, an informant-based delirium assessment tool. Specifically, the objectives were to train ChatGPT-3.5 and ChatGPT-4 to understand and correctly apply the Sour Seven Questionnaire to clinical vignettes using prompt engineering, assess the performance of these AI models in identifying and scoring delirium symptoms against scores from human experts, and refine and enhance the models’ interpretation and reporting accuracy through iterative prompt optimization.

**Methods:**

We used prompt engineering to train ChatGPT-3.5 and ChatGPT-4 models on the Sour Seven Questionnaire, a tool for assessing delirium through caregiver input. Prompt engineering is a methodology used to enhance the AI’s processing of inputs by meticulously structuring the prompts to improve accuracy and consistency in outputs. In this study, prompt engineering involved creating specific, structured commands that guided the AI models in understanding and applying the assessment tool’s criteria accurately to clinical vignettes. This approach also included designing prompts to explicitly instruct the AI on how to format its responses, ensuring they were consistent with clinical documentation standards.

**Results:**

Both ChatGPT models demonstrated promising proficiency in applying the Sour Seven Questionnaire to the vignettes, despite initial inconsistencies and errors. Performance notably improved through iterative prompt engineering, enhancing the models’ capacity to detect delirium symptoms and assign scores. Prompt optimizations included adjusting the scoring methodology to accept only definitive “Yes” or “No” responses, revising the evaluation prompt to mandate responses in a tabular format, and guiding the models to adhere to the 2 recommended actions specified in the Sour Seven Questionnaire.

**Conclusions:**

Our findings provide preliminary evidence supporting the potential utility of AI models such as ChatGPT in administering standardized clinical assessment tools. The results highlight the significance of context-specific training and prompt engineering in harnessing the full potential of these AI models for health care applications. Despite the encouraging results, broader generalizability and further validation in real-world settings warrant additional research.

## Introduction

### Background

Since its public launch in November 2022, OpenAI’s ChatGPT [1] has generated a significant global impact, with a potential to transform various sectors, including medical education, research, and practice [2]. ChatGPT leverages the power of deep learning to create human-like responses in natural language conversations. ChatGPT belongs to the suite of generative pretrained transformer (GPT) models and is one of the most sophisticated and expansive publicly accessible language models.

ChatGPT has been trained on a vast corpus of online text (570 GB or approximately 300 billion words), including open-source contents from websites, articles, and books collected up until the end of 2021 [3]. Currently, ChatGPT is available for public in 2 models: ChatGPT-3.5 and ChatGPT-4. ChatGPT-4, which is the latest model at the time of this study, benefits from a larger training data set, offering the potential for more accurate and reliable generation of human-like natural language text. A more in-depth discussion of the genesis and detailed training process of ChatGPT can be found in a recent review by Roumeliotis and Tselikas [3]. Unlike search engines, ChatGPT operates in a self-contained manner, meaning that it does not connect and search the internet to fetch real-time information when formulating responses. When a user interacts with ChatGPT, it does not actively scan through existing data nor does it directly duplicate preexisting information. Its responses are purely predictive, based on the patterns and structures that it has learned from its training data set.

ChatGPT’s potential applications in medical education are promising and manifold [4]. As a general purpose artificial intelligence (AI) system, ChatGPT can be applied to a wide range of tasks without substantial modification or fine-tuning. In a recent conversation between Eysenbach and ChatGPT [2], ChatGPT articulated its potential roles in medical education. These include tailoring resources to an individual learner’s needs; enhancing textbooks with additional visual aids to simplify complex medical concepts; designing and delivering medical curriculums; and creating and simulating virtual patient scenarios or clinical vignettes.

To explore ChatGPT’s potential in aiding the teaching of clinical decision-making as well as serving as a clinical decision support tool, several studies and evaluation projects have explored ChatGPT’s ability to diagnose or solve complex clinical vignettes. Hirosawa et al [5] examined the diagnostic accuracy of ChatGPT-3.5’s differential diagnosis lists for clinical vignettes involving common chief complaints. Their study entailed the creation of clinical vignettes by physicians, with ChatGPT-3.5 then used to formulate differential diagnoses for these vignettes. The diagnoses derived by ChatGPT-3.5 were compared with physicians’ diagnoses, assessing the AI’s diagnostic accuracy. Rao et al [6] assessed ChatGPT’s diagnostic accuracy across comprehensive clinical vignettes, considering patient age, gender, and severity of clinical presentation. They used the Merck Sharpe & Dohme Clinical Manual’s 36 published clinical vignettes, presenting differential diagnosis, diagnostic testing, final diagnosis, and management questions sequentially. They then analyzed ChatGPT’s performance on differential diagnosis, diagnostic testing, final diagnosis, and management based on the patient’s age, gender, and case severity. Levine et al [7] compared the diagnostic and triage abilities of ChatGPT-3 against those of laypeople and practicing physicians, using 48 validated case vignettes. Despite their innovative approach, they conceded that their method, which consisted of giving ChatGPT-3 a sample vignette as a prompt, did not adequately establish context or evaluate the model’s baseline understanding. van Bulck and Moons [8] evaluated ChatGPT’s understanding and response to complex clinical questions within the cardiovascular domain by analyzing the trustworthiness, value, and potential risks of ChatGPT-generated responses on 4 vignettes that simulated virtual patient questions, evaluated by 20 experts. Au Yeung et al [9] designed synthetic clinical histories in the style of vignettes and tasked ChatGPT with predicting the 5 most probable diagnoses. This approach helped assess the model’s ability to comprehend and generate diagnostic suggestions based on simulated clinical histories. Nevertheless, based on their evaluation results, they concluded that there is a lack of readiness of transformer-based chatbots for use as a patient-facing clinical tool in its current form.

These previous studies demonstrated the potential of ChatGPT in facilitating the teaching and learning of clinical decision-making and problem-solving using validated clinical vignettes. However, a gap remains in the current literature regarding how these AI models can be optimally trained to understand and effectively use validated assessment tools. Furthermore, no studies to date have evaluated AI’s performance, like ChatGPT’s, in responding to clinical vignettes after being trained to comprehend and apply a validated clinical assessment tool. Finally, none of these existing studies have incorporated content-specific training or attempted to optimize the outputs of ChatGPT models after evaluating its performance to mimic the workflow of a human assessor when completing a standardized assessment form.

Our research seeks to address this need by introducing a unique approach that begins with ensuring the AI model’s comprehension of delirium assessment and pretraining and validating its understanding of the Sour Seven Questionnaire. We chose the Sour Seven Questionnaire because of its brevity and coverage of the Confusion Assessment Method (CAM) [10] diagnostic features and applicability to patients who are nonverbal including those with aphasia and dementia [11]. The Sour Seven Questionnaire is specifically developed for use by nurses and untrained informal caregivers and has been validated against clinical assessment of geriatric psychiatrists. CAM is the most widely used delirium assessment tool and is developed for nonpsychiatrically trained clinicians to quickly assess delirium. There is a family version of CAM (FAM-CAM) [12], but it requires a longer administration time than Sour Seven. Moreover, prior research reported better sensitivity of Sour Seven than FAM-CAM (72.9% vs 54.1%) and reported that family caregivers preferred Sour Seven over FAM-CAM [13].

We opted to use a publicly available research article on the validation of the tool as the foundational content. Our method highlights the importance of pretraining and using simple prompt engineering strategies that can be used across a range of scenarios involving AI models such as ChatGPT. Prompt engineering refers to the process of thoughtfully crafting the input prompts to guide the AI model toward generating the desired output. This involves using specific keywords, context setting, and explicit instructions for the type of response required. We demonstrate how to effectively pretrain and validate these models to comprehend and use validated clinical tools, such as the Sour Seven Questionnaire, in their assessments using prompt engineering. This structured approach emphasizes simplicity and accessibility, making it possible for a wide array of individuals, including those without clinical or engineering backgrounds, to effectively use AI models in assessing clinical vignettes.

### Objective

In this exploratory study, we sought to evaluate the performance of ChatGPT models on assessing delirium in validated case vignettes after receiving content-specific training on a standardized assessment tool, the Sour Seven Questionnaire [11]. After training ChatGPT on administration of the Sour Seven Questionnaire, we compared its performance in the following areas against 5 research investigators who served as human expert assessors: (1) identification of the presence or absence of delirium; (2) the Sour Seven scoring patterns; and (3) the use of information from each case vignette to inform positive Sour Seven items and the total Sour Seven score. We then applied a prompt engineering approach to optimize the reporting and interpretation of the delirium assessment outcomes. Different from the application of ChatGPT as a tool for teaching and facilitating clinical decision-making for medical professionals only, the current application of ChatGPT falls within the domain of patient and consumer education, and more specifically, family delirium education. This is because early detection and differential diagnosis is challenging because both delirium and dementia cause cognitive impairments and often share similar symptoms (hallucinations, delusions, language impairment, etc) [14,15]. Furthermore, clinicians often lack information about the patient’s baseline cognitive status required to determine whether the cognitive change or impairment is acute (delirium) or chronic (dementia) [15,16]. To have a better understanding of the patient’s baseline cognitive status, experts advocate for the engagement of family and friends (informal caregivers), who know the patient best, to be part of the clinical decision-making and assist with early detection, prevention, and management of delirium in older adults with and without dementia [17-21].

## Methods

### Design

We conducted an exploratory study using content-specific training and prompt engineering strategies to iteratively evaluate and optimize the performance of ChatGPT models on interpreting and reporting of delirium assessment outcomes in standardized clinical vignettes.

### Materials

#### Delirium Assessment Tool: The Sour Seven Questionnaire

The Sour Seven Questionnaire [11] was originally developed for informal caregivers and untrained nurses to assess delirium in hospitalized older adults. The Sour Seven Questionnaire consists of 7 yes or no items, describing 4 symptoms that align with the *Diagnostic and Statistical Manual of Mental Disorders, Fourth and Fifth Edition* diagnostic criteria and CAM [10] (ie, disturbances in awareness, disturbances in attentiveness, fluctuations in awareness and attentiveness, and disordered thinking), plus 3 additional items to assess disorganized behavior, unexplained impairment in eating or drinking, and unexplained impairment in mobility or movement.

A weighted score of 4 suggests “possible delirium” (positive predictive value 89%; sensitivity 89.5%; specificity 90%) and a weighted score of 9 suggests “delirium” (positive predictive value 100%; sensitivity 63.2%; specificity 100%) [11].

#### Clinical Case Vignettes

The 5 clinical case vignettes used in this study were standardized and previously validated for use in geriatric nursing education research [22,23]. Each case vignette simulated hospital bedside interactions between nursing staff, family, and an older adult who demonstrated signs and symptoms of one of the following conditions: (1) hyperactive delirium, (2) hypoactive delirium, (3) dementia, (4) hyperactive delirium superimposed on dementia, and (5) hypoactive delirium superimposed on dementia. These vignettes were previously developed as a way to assess health care staff knowledge about delirium and the motoric subtypes of delirium in the hospital setting. The vignettes were evaluated by a panel of health care providers with geriatric and psychiatric expertise. The panel independently rated the diagnosis and delirium subtype (where appropriate) for each vignette; their overall agreement on the cases was 84% and their agreement on the identification of the delirium motoric subtypes was 100%. They were further refined by McCrow et al [23] and tested on nursing home staff [24]. In this study, we used the version of the 5 vignettes that simulated hospital bedside interactions [23]. We did not modify the content of the vignettes, but we presented them in 2 formats either as an unstructured paragraph or a structured paragraph in which each sentence was assigned a number.

### Procedures

#### Overview

The iterative evaluation, validation, and optimization process used in this exploratory study consisted of the following main steps: (1) ChatGPT baseline knowledge assessment; (2) content-specific training and preliminary validation; (3) pilot evaluation; (4) evaluation of ChatGPT models against human experts; and (5) ChatGPT output optimization. Steps 1 to 3 were first tested in ChatGPT-3.5. After validation or evaluation of steps 1 to 3, steps 4 and 5 were then tested in both ChatGPT-3.5 and ChatGPT-4 models. A new ChatGPT session was started to test step 4 and step 5, respectively. In each new session, steps 1 and 2 had to be repeated as a primer; that is, when testing step 4 in a new session, the actual process was step 1 followed by step 2 then step 4. Similarly, when testing step 5 in a new session, the process was step 1 followed by step 2 then step 5.

#### Step 1: Assessment of Baseline Knowledge

To assess ChatGPT’s baseline knowledge of the Sour Seven Questionnaire, we asked the ChatGPT, “What are the sour-seven questionnaire items and how do you score them?” The response showed that ChatGPT did not have existing knowledge of this assessment tool (“I’m sorry for any confusion, but as of my training cut-off in September 2021, I don’t have detailed information on the specific items or scoring system used in the Sour-Seven Questionnaire for Delirium Assessment.”). This suggested that additional content-specific training was required.

#### Step 2: Content-Specific Training and Preliminary Validation

To provide content-specific training, we segmented the entire text of the published manuscript of the original validation study of the Sour Seven Questionnaire [11] into portions that conformed to ChatGPT’s input token size limit. The segmented text blocks were then fed to ChatGPT with the context-setting prompt, “Here is a research article describing the Sour Seven assessment instrument broken down to series of text blocks. Here is the first text block.” This process was repeated until all text blocks had been input into the model. This context was essential in guiding the model’s understanding of the text it was processing. All images and portions of the text formatted as tables in the manuscript had to be removed because ChatGPT could only accept text-based input. Through this training process, ChatGPT was expected to understand the individual components of the Sour Seven Questionnaire and their relevance in delirium detection. ChatGPT’s understanding of the Sour Seven Questionnaire was then validated using the following prompt, “What is the Sour-Seven Questionnaire for Delirium?” Subsequently, we instructed ChatGPT on the scoring criteria of each individual Sour Seven item. For example, for the first item, “Altered level of awareness to the environment in any way different than being normally awake,” the model was prompted with “If ‘Yes’ to ‘Altered level of awareness to the environment in any way different than being normally awake,’ give a score of 3. If ‘No,’ give a score of 0.” This process was repeated for each of the items in the Sour Seven Questionnaire. Then, we instructed the ChatGPT on how to interpret the Sour Seven total score using the following scoring criteria available in the Sour Seven Questionnaire, “A score of 4 or higher indicates ‘possible delirium’ The suggested course of action is to evaluate potential medical causes, meds/substances. A score of 9 or higher indicates ‘delirium’ and warrants immediate medical evaluation.” To verify the model’s comprehension and integration of the scoring criteria, we used a validation prompt: “What is the Sour Seven scoring criteria?” After validating the understanding of ChatGPT, we proceeded to test ChatGPT’s ability to score standardized clinical vignettes and identify the presence or absence of delirium symptoms using the Sour Seven Questionnaire.

#### Step 3: Pilot Evaluation

To test ChatGPT’s ability to apply the Sour Seven Questionnaire to standardized clinical vignettes, we performed 3 pilot trials using ChatGPT-3.5 on each of the 5 clinical vignettes. Each vignette was fed to the ChatGPT as an unstructured paragraph after the prompt, “Here is a scenario. please evaluate using sour seven questionnaire items and provide the scoring breakdown.” The output from the ChatGPT model was regenerated using ChatGPT’s “regenerate” button 2 more times for each vignette (3 pilot trials per vignette × 5 vignettes = 15 pilot trials).

#### Step 4: Evaluation of ChatGPT Model Performance Against Human Experts

To compare the performance between ChatGPT models and human experts, we further structured the case vignettes by numbering each sentence in the case vignette (see Textbox 1 for the structured case vignette prompt) and created a standardized clinical vignette evaluation form for human experts to complete (Multimedia Appendix 1). The output from the ChatGPT model was regenerated using ChatGPT’s “regenerate” button 4 more times for each vignette (=5 trials per vignette × 5 vignettes=25 trials).

The human experts consisted of 4 interdisciplinary clinician researchers with MD and RN degrees (DF, SL, PS, and RS) and one health sciences researcher (SL); all had expertise in delirium, dementia, or delirium superimposed on dementia research. All experts received the standardized evaluation form along with a PDF copy of the original Sour Seven scale over email. Each expert completed the evaluation form independently. No training was provided to experts on how to complete the Sour Seven Questionnaire or the evaluation form. This was to simulate the real-world use of the Sour Seven Questionnaire, that is, being completed by untrained informants. After all experts submitted the evaluation form, they received an anonymized summary of the scoring results of all experts and were asked to provide feedback on variations that they noticed in the summary.

**Validation question 1 (prompt) and ChatGPT’s response.**

**Prompt**

- “What is the Sour-Seven Questionnaire for Delirium Assessment?”

**ChatGPTresponse**:

- “Yes, the Sour Seven Delirium Screening tool is typically administered as a questionnaire that asks the caregiver or healthcare provider to observe the individual and answer questions about their behavior and symptoms. The tool starts with a general question asking the caregiver or healthcare provider if they have observed any of the following during their interaction with the individual”:
- “During your interaction with the person today, have you observed any of the following?”
- The Sour Seven questions follow this general question and are designed to assess the individual’s cognitive function and behavior. The caregiver or healthcare provider answers each of the seven questions based on their observation of the individual’s behavior and symptoms during the interaction.

**The Sour Seven questions are:**

1. Altered level of awareness to the environment in any way different than being normally awake.
2. Reduced attentiveness; inability to focus on you during the interaction.
3. Fluctuation in awareness and attentiveness, such as drifting in and out during an interaction or through the day.
4. Disordered thinking; the response (whether verbal or action) is unrelated to the question or request.
5. Disorganized behavior; purposeless, irrational, under-responsive, or over-responsive to requests.
6. Unexplained impaired eating or drinking (excluding appetite); unable to perform the actions to feed oneself.
7. Unexplained difficulty with mobility or movement.

- Based on the caregiver or healthcare provider’s answers to these questions, a score is calculated and used to determine if the individual is at risk for delirium. If the score is positive, further evaluation and diagnostic testing may be needed to confirm the diagnosis of delirium and identify any underlying causes.”

#### Step 5: Prompt Optimization

After comparing results between ChatGPT models and human experts, we iteratively tested additional prompts to reduce inconsistencies and errors in ChatGPT outputs as well as standardize the format of the output into a table format similar to the evaluation form for the human experts. The final set of prompts used to optimize the ChatGPT outputs is available in Multimedia Appendix 2.

### Data Analysis

We performed descriptive statistics in Excel (Microsoft Corporation) to summarize the scoring patterns between ChatGPT models and those of human experts.

### Ethical Considerations

Our study did not involve human participation beyond the authors who contributed to writing the prompts (YC) and providing expert reviews of the Sour Seven assessment on clinical vignettes (SYL, DMF, RS, SL, and PS). After consultation with the University of Pittsburgh Institutional Review Board, it was determined that an ethical review was not necessary for this study.

## Results

### Content-Specific Training and Preliminary Validation

The initial content-specific training on Sour Seven was successful. After training, ChatGPT was able to describe the Sour Seven Questionnaire (Textbox 1) and its scoring criteria (Textbox 2) in response to the validation prompts.

**Validation question 2 (prompt) and ChatGPT’s response.**

**Prompt**

- “What is sour seven scoring criteria?”

**ChatGPT response**

- “The Sour Seven scoring criteria are as follows:

1. Altered level of awareness to the environment in any way different than being normally awake: 3 points for “yes,” 0 points for “no”
2. Reduced attentiveness; inability to focus on you during the interaction: 4 points for “yes,” 0 points for “no”
3. Fluctuation in awareness and attentiveness, such as drifting in and out during an interaction or through the day: 3 points for “yes,” 0 points for “no”
4. Disordered thinking; the response (whether verbal or action) is unrelated to the question or request: 3 points for “yes,” 0 points for “no”
5. Disorganized behavior; purposeless, irrational, under-responsive or over-responsive to requests: 2 points for “yes,” 0 points for “no”
6. Unexplained impaired eating or drinking (excluding appetite); unable to perform the actions to feed oneself: 2 points for “yes,” 0 points for “no”
7. Unexplained difficulty with mobility or movement: 1 point for “yes,” 0 points for “no”

- The maximum possible score on the Sour Seven questionnaire is 18. Scores of 4 or above may indicate possible delirium, while scores of 9 or above may indicate delirium and warrant immediate medical evaluation. However, it is important to note that the Sour Seven questionnaire is just a screening tool and a positive screen should be followed up by a more comprehensive evaluation by a healthcare professional.”

### Pilot Evaluation

The pilot evaluation was conducted across 15 trials using ChatGPT-3.5. When ChatGPT-3.5 determined a Sour Seven item was positive, it included “Yes” in the response and a rationale to justify the score across all 15 trials (eg, “Yes, as Charles is not able to answer appropriately and is repeating responses. Score of 3.”). However, when ChatGPT-3.5 determined a Sour Seven item was “not positive,” it did not always include an explicit “No” in the response (eg, “Not specified in the scenario. Score of 0”). An examination of ChatGPT’s interpretation of the Sour Seven total scores also revealed additional issues:

1. An out-of-range cutoff score: the 2 Sour Seven cutoff scores were 4 and 9, but ChatGPT used 12 as the cutoff score on one occasion when the total score was 15 (“Charles meets the criteria for delirium with a score of at least 12”)
2. Incorrect cutoff scores (ChatGPT sometimes used 4 as the cutoff score when the total score was above 9)
3. Missing an explicit total score (eg, the ChatGPT provided “(3+4+3+3+2)” instead of “15”)
4. Incorrect interpretation of the Sour Seven total score (a score ≥9 should suggest “delirium,” but sometimes ChatGPT interpreted it as “possible delirium”)

### Evaluation of ChatGPT Model Performance Against Human Experts

Of the 5 clinical vignettes, the first 4 depicted different types of delirium while the last vignette described dementia without delirium (Table 1). ChatGPT-3.5, ChatGPT-4, and human experts all gave a total score above 9 (“delirium”) for the first 4 clinical vignettes across all trials and a total score below 4 (the lower bound cutoff score of “possible delirium”) for the last vignette across all trials. This means that ChatGPT models and human experts all accurately gave a total score that would suggest “delirium” for vignettes that portrayed some type of delirium and a total score that would not suggest delirium for the vignette that depicted dementia without delirium. The variation in the total score across ChatGPT trials were smaller than the variation between human experts (as shown by smaller SDs and ranges) for most vignettes.

**Table 1:** Sour Seven total scoring variation—ChatGPT-3.5, ChatGPT-4, and human experts across 5 case vignettes^a^.

	ChatGPT-3.5: across 5 trials		ChatGPT-4: across 5 trials			Human experts: across 5 trials	
	Values, mean (SD); range	Diff^b^	Values, mean (SD); range	Diff	Values, mean (SD)		Values, median (range)
Vignette 1: hypoactive delirium	13.8 (1.5); 12-15	–0.2	15 (0); —^c^ (the total score was 15 in all 5 trials)	1	14 (1.7)		14 (12-17)
Vignette 2: hyperactive delirium	12 (0); — (the total score was 12 in all 5 trials)	–2.4	15 (0); — (the total score was 15 in all 5 trials)	0.6	14.4 (1.2)		15 (12-15)
Vignette 3: hypoactive delirium superimposed on dementia	12.4 (0.8); 12-14	–3.4	15.4 (1.5); 13-17	–0.4	15.8 (1.6)		17 (13-17)
Vignette 4: hyperactive delirium superimposed on dementia	12.2 (1.6); 10-15	–1	15 (0); — (the total score was 15 in all 5 trials)	1.8	13.2 (1.5);		12 (12-15)
Vignette 5: dementia	2 (0); — (the total score was 2 in all 5 trials)	1.2	1 (0.9); 0-2	0.2	0.8 (1)		0 (0-2)

^a^Sour Seven total score: ≥4 suggests “possible delirium: evaluate potential medical causes, meds/substances”; ≥9 suggests “delirium: immediate medical evaluation required.”

^b^Diff: difference in mean scores (mean total score of the ChatGPT trials–mean total score of 5 human experts).

^c^Not applicable.

An examination of what contents in each vignette informed the assignment of a positive score to Sour Seven Questionnaire items showed that in most ChatGPT trials, the specific sentences used by the ChatGPT models overlapped with those used by human experts (see Multimedia Appendix 3 for more details). However, in 2 out of the 5 cases (vignettes 2 and 4), ChatGPT models used one extra sentence in each case to inform positive Sour Seven scores that were not used by any human experts. Further analysis of these instances revealed that ChatGPT misapplied these sentences due to a misunderstanding of their context. For instance, in vignette 4, sentence 7 (“After his uneventful surgery he had some slight confusion and memory problems which were *similar to his admission observations*”) was mistakenly used to indicate fluctuations in awareness and attentiveness, a symptom assessed by item 3 of the Sour Seven Questionnaire. Interestingly, while the ChatGPT models may assign the same total score across multiple trials of the same vignette, the sentences from the vignette used to justify its decisions sometimes varied (see Multimedia Appendix 3 for more details).

Regarding narrative responses and score interpretation, we observed the following differences:

1. Response style: ChatGPT-3.5 provided succinct, single-sentence explanations for each scored item, while ChatGPT-4 gave detailed, multisentence narratives (Multimedia Appendix 2).
2. Treatment of 0 scores: ChatGPT-3.5 simply noted a lack of relevant information for items scored as 0, while ChatGPT-4 offered explicit reasoning.
3. Evidence presentation: ChatGPT-4 used verbatim excerpts from case studies as evidence, while ChatGPT-3.5 summarized the relevant content.
4. Assignment of partial scores: ChatGPT-4, in cases of uncertainty, occasionally assigned a score of 1 (when the possible scores were 0 or 2).
5. Scoring cutoffs and interpretation: ChatGPT-3.5 was able to recite the 2 cutoff scores (4 and 9) and the respective interpretations (“possible delirium” and “delirium”), it still did not select the accurate cutoff score consistently and sometimes still refer to the lower cutoff score of 4 when the total Sour Seven score was above 9. ChatGPT-4 chose to use the language “high likelihood of delirium” for scores of 9 and above.
6. No recommended actions: ChatGPT-4 primarily provided interpretations, such as “high likelihood of delirium,” but neglected to suggest the next steps contained within the Sour Seven assessment form (ie, warranting immediate medical evaluation or attention).

**ChatGPT Prompt Optimization**

We optimized ChatGPT’s performance through several iterations targeting specific areas:

1. Scoring methodology: we adjusted the training prompt to ensure that only definitive “yes” or “no” responses were accepted for each item, ruling out intermediate or partially affirmative responses. The prompt used was “Only a definitive Yes or No response is accepted for each item; No intermediate or partially affirmative responses are accepted.”
2. Output format: the evaluation prompt was revised to mandate responses in a table format.
3. Restrict recommended actions to only the 2 actions specified in the published Sour Seven Questionnaire Scoring Instruction [11]: the evaluation prompt was further revised to instruct ChatGPT to adhere strictly to only the 2 possible follow-up actions specified in the published assessment form, that is, “evaluate potential medical causes, meds/substances” and “immediate medical evaluation,” as shown in Textbox 3. This step was to explore the feasibility of preventing ChatGPT from giving medical advice not vetted by a medical professional.

With the modified prompts, both ChatGPT-3.5 and ChatGPT-4 models successfully returned responses in the required tabular format, and each accurately followed the scoring interpretations consistent with the instruction in the published Sour Seven Questionnaire form. A substantial change was observed in ChatGPT-3.5, which rectified its earlier issue of misapplying scoring cutoff points and began to correctly implement them in its evaluations. However, an instance of total score miscalculation was noted, where ChatGPT-3.5 incorrectly totaled item scores to 17 instead of the correct 15.

The scoring performance remained consistent for both models after prompt optimization, with ChatGPT-4 exhibiting less variability. ChatGPT-3.5 showed one instance of case 4 where it generated a score of 7 where the other 4 instances generated a score of 13.

**Modified evaluation prompt.**

**Prompt**

- Please evaluate the following scenario using the Sour Seven Questionnaire and provide the scoring breakdown and reasoning for each item. The sentences are numbered for your reference. Please evaluate using sour seven questionnaire items and provide the scoring breakdown and reasoning. For each observation, please specify the sentence number(s) that informed your decision.
- Scenario: [insert case vignette]
- Please construct your response in the table format as follows.
- Sour Seven Item | Response (Yes/No) and (Score of X) | Sentence numbers | Reasoning |
- Total Score should be added at the bottom row of the table.
- After that, please provide the suggested next steps based on the following Sour Seven scoring interpretation:
- If the total score is less than 4, it suggests less likelihood of delirium. In this case, no follow-up actions need to be suggested.
- If the total score falls between 4 and 8, it indicates “possible delirium.” The suggested course of action is to “evaluate potential medical causes, meds/substances.”
- If the total score is 9 or higher, it indicates “delirium.” This warrants “immediate medical evaluation.”
- Please provide the relevant interpretation only.
- Do not provide any further recommendations beyond this scope.”

## Discussion

### Principal Findings

In this study, we applied context-specific training and prompt engineering to instruct ChatGPT on how to interpret and apply the Sour Seven Questionnaire to validated clinical vignettes.

We found that both ChatGPT-4 and ChatGPT-3.5 demonstrated consistent abilities in identifying and scoring delirium across a diverse set of vignette cases over 5 trials, with total scores and ranges comparable to those of human experts. Notably, ChatGPT-4 outperformed ChatGPT-3.5 with regard to lower variability and closer alignment with human expert scoring.

However, despite their overall performance aligning considerably with human expert evaluations, our analysis revealed specific areas of concern and opportunities for further refinement. For instance, although the total scores may correctly identify delirium in a manner similar to human experts, there were a few cases where ChatGPT incorrectly applied sentences for its reasoning process (see Multimedia Appendix 3 for more details). This highlights potential challenges concerning the model’s ability to comprehend and use complex context within its decision-making process and underline potential weaknesses of the model in interpreting nuances in clinical presentations. These results emphasize the importance of rigorous validation procedures when using AI models in health care and medical education [25].

In addition, before prompt optimization, ChatGPT-3.5 demonstrated difficulties with accurately applying scoring cutoff points and deviated from the guidelines of the Sour Seven Questionnaire. This suggests that while the AI model can comprehend and apply rules-based scoring, they may encounter issues when dealing with precise thresholds if these are not articulated in a straightforward manner. Similarly, ChatGPT-4, on the other hand, revealed a tendency to assign a midpoint score of 1 in uncertain or ambiguous situations, although the original assessment tool only permits binary scoring options (ie, 0 or 2). Interestingly, this behavior reflects a more human-like approach to decision-making, allowing for a more nuanced understanding of symptom manifestation. However, it also signifies a departure from the strict guidelines of the clinical assessment tool. While this capacity to capture nuances could be beneficial in certain contexts, it could lead to discrepancies when applied to strict clinical guidelines, potentially resulting in over- or underassessment of patient conditions.

These findings emphasize the crucial role of iterative prompt optimization in improving the performance of ChatGPT models [26]. The use of straightforward prompt engineering strategies, such as the use of simple and clear instructions, structuring input data in a certain manner, and formatting the output to comply with expected standards, can markedly enhance the accuracy and usability of ChatGPT.

### Implications for Medical Education

ChatGPT can play a crucial role in medical education, particularly in addressing complex conditions such as delirium. Despite its serious implications, delirium often remains underrecognized or misdiagnosed and inadequately managed within health care settings [27]. Past research shows that these gaps in recognizing or adequately managing delirium arises partially from a lack of necessary knowledge and confidence among health care professionals in screening for and diagnosing delirium and delirium superimposed on dementia [28,29]. Unfortunately, medical and nursing education often does not place enough emphasis on delirium, leaving practitioners underprepared to identify and manage this complex condition effectively [14].

Leveraging the vast knowledge base and natural language processing capabilities, ChatGPT can engage nursing and medical students and practitioners in interactive conversations to enhance their understanding of and confidence in recognizing delirium. From recognizing risk factors and differentiating subtypes to grasping diagnostic criteria and management strategies, ChatGPT provides valuable insights and guidance. Its ability to simulate diverse patient scenarios and offer evidence-based recommendations can contribute to a more comprehensive and immersive learning experience, ultimately empowering nursing and medical students to deliver improved care for patients experiencing delirium [30,31]. Finally, future studies can build on the current work, modifying the content-specific training and prompt engineering strategies reported herein to optimize the teaching, learning, and administration of clinician-administered delirium assessment tools (eg, CAM), as well as exploring the use of ChatGPT as a clinical decision support system to improve medical professionals’ confidence in differential diagnosis of delirium, dementia, and delirium superimposed on dementia.

### Implications for Family Delirium Education

To aid early detection of delirium superimposed on dementia, family and friends not only need education on delirium, dementia, and delirium superimposed on dementia but also need training and tools to assess and report delirium symptoms in their care recipients systematically and reliably. ChatGPT’s application in the context of family delirium education could have a significant impact on patient care, particularly in augmenting family caregiver education for delirium detection and management. The current work contributes to this important area of family education by confirming ChatGPT’s ability to complete a standardized informant-based delirium assessment form in a manner mimicking what a human assessor would, as well as providing consistent interpretation of the assessment scores and recommended follow-up actions. Using AI in this manner can not only amplify caregivers’ knowledge about delirium, but it can also serve as a practical tool in real-life caregiving situations. Future work should evaluate feasibility and acceptability of using ChatGPT as a virtual teaching assistant to guide family caregivers in completing standardized informant-based delirium assessment forms, enabling them to monitor and communicate more effectively with their health professionals about acute changes in their care recipient.

### Comparisons With Prior Work

Several recent studies have explored the performance of ChatGPT in various clinical contexts, demonstrating its ability to provide differential diagnoses based on clinical vignettes. For instance, Hirosawa et al [5] assessed ChatGPT’s diagnostic accuracy across an array of case vignettes representing common chief complaints. They used a single, straightforward prompt (“tell me the top ten suspected illnesses for the following symptoms:”) to elicit this information, and the results showcased significant accuracy.

A study by Rao et al [6] delved deeper into ChatGPT’s potential examining its capacity to support comprehensive iterative clinical reasoning through sequential prompts, demonstrating ChatGPT’s ability to incorporate information from earlier parts of a conversation into downstream responses. However, these studies did not explore the benefits of context-specific training of ChatGPT using prompt engineering, aiming to guide the model to administer an assessment tool or modify its behavior in a particular manner, as we have demonstrated in our study. This novel approach substantially enhanced the ChatGPT model’s capabilities, yielding more precise and reliable results. In addition, our work goes beyond the focus on health care professionals and clinical settings by training ChatGPT on an informant-based assessment tool.

Therefore, our research presents a unique approach and contributes novel insights into how ChatGPT can be optimally trained and used in various health care scenarios, including lay users, such as family caregivers in nonclinical settings.

### Limitations

The potential application of ChatGPT in health care settings, as demonstrated in this study, is indeed promising. However, acknowledging the inherent limitations in the model is crucial. Both the ChatGPT-3.5 and ChatGPT-4 models were trained on data only up to September 2021, meaning they may not reflect more recent developments or findings.

A significant challenge with AI models, including ChatGPT, is the potential for AI hallucinations, where the AI system generates plausible but factually incorrect responses [32]. Moreover, similar to any AI model, ChatGPT is not immune to biases, either explicit or implicit, that might have been present in the training data. These biases can manifest in the outputs, which could potentially lead to skewed or inaccurate information [33]. Another technical limitation is the lack of guaranteed replicability of responses in the ChatGPT models, potentially introducing variability in outputs despite identical inputs. Regarding study-specific limitations, the focus on one informant-based assessment tool for delirium detection and the relatively small number of trials and human experts involved could limit the generalizability of our findings. To comprehensively evaluate ChatGPT’s effectiveness in learning and administering clinical assessment tools, further research involving larger data sets, diverse clinical contexts, and a variety of assessment tools is warranted. This study used validated clinical vignettes for privacy concerns of feeding real patient data or clinical case. Future research could investigate incorporating real patient data or user testing with family caregivers, provided the ethical and privacy considerations are adequately addressed.

### Implications for Future Generative AI and ChatGPT Research

Our research underscores several crucial directions for future AI research for health care and medical education, particularly with models such as ChatGPT. It highlights the potential benefits of context-specific training to enhance models’ domain proficiency, thereby improving their performance in identifying specific conditions such as delirium. Prompt engineering also emerges as a key factor in enhancing AI utility [26]. By thoughtfully adjusting model prompts, we can improve the precision, relevance, and clarity of model outputs.

### Conclusions

This exploratory study provides substantial evidence of the effectiveness of AI models, particularly ChatGPT, in learning and applying standardized informant-based delirium assessment tools such as the Sour Seven Questionnaire. Our findings demonstrate that with context-specific training and prompt engineering, the performance and utility of ChatGPT significantly improved in detecting and scoring delirium. These results closely aligned with evaluations performed by human experts, highlighting the potential of AI in health care, specifically in managing complex conditions such as delirium. The implications of our findings extend beyond professional health care settings and carry significant potential for aiding family caregivers in nonclinical environments. Our results suggest that ChatGPT can be a valuable tool in educating family caregivers about delirium, providing them with the knowledge necessary to identify early signs of onset or progression. This could allow them to effectively communicate these changes to health care professionals, facilitating timely intervention and improved patient outcomes.

We used validated clinical vignettes as the basis for evaluating the performance of the trained ChatGPT models in this study. While our findings are encouraging, their generalizability to other clinical scenarios or different assessment tools necessitates further exploration. Future research must encompass larger-scale studies involving diverse clinical contexts and real-world settings that include ChatGPT interactions with clinicians and family caregivers to validate and generalize our results. Ethical considerations, such as the use of real patient data for AI training, remain an important aspect of future investigations.

## Appendix

Multimedia Appendix 1 Standardized clinical vignette evaluation form completed by human experts, used for comparison with ChatGPT model performance in structured case vignette assessments.

Multimedia Appendix 2 Exemplars of author prompts and verbatim outputs by ChatGPT-3.5 and ChatGPT-4.

Multimedia Appendix 3 Contents of each vignette used to identify any positive Sour Seven items.

## Abbreviations

AI: artificial intelligence
CAM: Confusion Assessment Method
FAM-CAM: family version of Confusion Assessment Method
GPT: generative pretrained transformer
