# Supplementary material for: Optimizing ChatGPT’s Interpretation and Reporting of Delirium Assessment Outcomes: Exploratory Study
Source: JMIR Form Res. 2024 Oct 1;8:e51383. doi: 10.2196/51383 (PMC11480687; doi:10.2196/51383)
Supplement: Multimedia Appendix 1 [file formative_v8i1e51383_app1.docx]

**Case 1: Anne**

| **No.^a^** | **Case vignette description^b^** |
| --- | --- |
| **1** | **Anne** is a 75 year old who has been transferred from another hospital to the coronary care unit at your acute care hospital. |
| **2** | Seven days ago she sustained a fractured neck of femur which required corrective surgery. |
| **3** | She wears glasses, uses a hearing aid and has a previous medical history of hypertension, type 2 diabetes and high cholesterol for which she is taking multiple medications. |
| **4** | She had an uneventful recovery until two days after her surgery when she had a myocardial infarction and consequently was transferred to your facility for coronary artery bypass surgery. |
| **5** | On admission to your facility her cardiac condition had stabilized however the pain from her hip surgical site continued to be a problem despite receiving oxycodone regularly every 4 h for at least the preceding 24 h. |
| **6** | Prior to her hospitalization Anne lived with husband, was independent in activities of daily living, drove a motor vehicle, played card games and went to bingo with her friends regularly. |
| **7** | During the hospital admission interview, Anne did not answer questions appropriately and repeatedly dozed off while being interviewed. |
| **8** | She appeared to be speaking to herself however you could not understand what was being said. |
| **9** | When questioned she didn't know that she was in hospital and thought that the year was 1998 instead of the correct year, 2009. |
| **10** | Additionally nursing staff reported that she needed to be continuously prompted to wake whilst she is being fed. |
| **11** | There was no mention in the medical notes from the referring hospital about any previous confusion. |

^a^ Each sentence in the case description is numbered for easy reference.

^b^ This case vignette was originally created and validated for geriatric nursing education research.^1^

Please evaluate this case using the Sour Seven Questionnaire^2^ below and indicate whether each of the seven observations is present (Yes/No). If you select **Yes**, please specify the sentence number(s) that informed your decision. If there are no relevant case descriptions, please select **No** and enter “NA” for the sentence number. You can use the **Comments (Optional)** column to provide more information about your reasoning or share any additional thoughts.

| Sour Seven Items | Yes | No | Sentence #, e.g., 1, 2, 11, etc. | Comments (Optional) |
| --- | --- | --- | --- | --- |
| Altered level of awareness to the environment in any way different than being normally awake. | 3 points | 0 point | 7, 10 | Repeated dozing – not just sleepy |
| Reduced attentiveness; inability to focus on you during the interaction | 4 points | 0 point | 7,10 |  |
| Fluctuation in awareness and attentiveness, such as drifting in and out during an interaction or through the day. | 3 points | 0 point |  | Though awareness is down seems across day plus not fluctuating |
| Disordered thinking; the response (whether verbal or action) is unrelated to the  question or request. | 3 points | 0 point | 7,8 |  |
| Disorganized behaviour; purposeless, irrational, under-responsive or over-responsive to requests. | 2 points | 0 point | 8 |  |
| Unexplained impaired eating or drinking (excluding appetite); unable to perform the actions to feed oneself. | 2 points | 0 point | 10 | Not as clear but is “being fed” |
| Unexplained difficulty with mobility or movement. | 1 point | 0 point |  |  |
| Total Score ^a^ | 14 **Points** | | | |

^a^ Total score: >=4 suggests “possible delirium: evaluate potential medical causes, meds/substances”; 9>= suggests “delirium: immediate medical evaluation required”

**Case 2: Charles**

| **No.^a^** | **Case vignette description^b^** |
| --- | --- |
| **1** | **Charles**is a 74 year old retired butcher who lives by himself in a one bedroom apartment, is independent, and drives a car. |
| **2** | He is on some blood pressure, cardiac and cholesterol medications. |
| **3** | He is admitted to hospital for a severe kidney infection with associated vomiting and severe pelvic and back pain and dehydration. |
| **4** | On admission he was commenced on intravenous therapy and narcotic pain medications. |
| **5** | Whilst in hospital he was reported to be sleeping poorly at night but during the day he had been pleasant and cheerful, though tired. |
| **6** | Additionally he displayed slight confusion as times although he was coherent the majority of the time. |
| **7** | Suddenly on the second night when you enter his room he is pacing, waving his arms in the air, and mumbling to himself. |
| **8** | He appears anxious and is verbally abusive to other people in the room. |
| **9** | When speaking to him you found it difficult to gain his attention and he was not able to engage in a sensible conversation. |
| **10** | When you asked questions he did not answer appropriately and repeated responses. |

^a^ Each sentence in the case description is numbered for easy reference.

^b^ This case vignette was originally created and validated for geriatric nursing education research.^1^

Please evaluate this case using the Sour Seven Questionnaire^2^ below and indicate whether each of the seven observations is present (Yes/No). If you select **Yes**, please specify the sentence number(s) that informed your decision. If there are no relevant case descriptions, please select **No** and enter “NA” for the sentence number. You can use the **Comments (Optional)** column to provide more information about your reasoning or share any additional thoughts.

| Sour Seven Items | Yes | No | Sentence #, e.g., 1, 2, 11, etc. | Comments (Optional) |
| --- | --- | --- | --- | --- |
| Altered level of awareness to the environment in any way different than being normally awake. | 3 points | 0 point | 7 | hyper |
| Reduced attentiveness; inability to focus on you during the interaction | 4 points | 0 point | 9 |  |
| Fluctuation in awareness and attentiveness, such as drifting in and out during an interaction or through the day. | 3 points | 0 point | 7,8,9 | inattention - |
| Disordered thinking; the response (whether verbal or action) is unrelated to the  question or request. | 3 points | 0 point | 7,8 |  |
| Disorganized behaviour; purposeless, irrational, under-responsive or over-responsive to requests. | 2 points | 0 point | 7,8 |  |
| Unexplained impaired eating or drinking (excluding appetite); unable to perform the actions to feed oneself. | 2 points | 0 point |  | These two are unclear to me. |
| Unexplained difficulty with mobility or movement. | 1 point | 0 point |  | These two are unclear to me. |
| Total Score ^a^ | 15 **Points** | | | |

^a^ Total score: >=4 suggests “possible delirium: evaluate potential medical causes, meds/substances”; 9>= suggests “delirium: immediate medical evaluation required”

**Case 3: Diane**

| **No.^a^** | **Case vignette description^b^** |
| --- | --- |
| **1** | **Dianne**a 79 year old lady was admitted to your hospital three days previously from a residential aged care facility following a myocardial infarction. |
| **2** | She appeared to be recovering and all her pathology and vital signs were within normal limits, however when you approached her today she was sleepy and other staff reported that she was not interested in eating or drinking. |
| **3** | Her family reported that she is not normally this sleepy and usually eats well. |
| **4** | When questioned about depression the family stated that she had no history of depression but her memory had been gradually getting worse. |
| **5** | They claimed that over the last 12 months she has slowly become less able to find her way around the facility and she forgets conversations they have had with her. |
| **6** | At times she accused them of stealing her car of which she had sold when she moved into the nursing home. |
| **7** | Diane is polite but sleepy when you approach her. |
| **8** | She does not recognize you as a nurse and thinks that you are her daughter, whom you do not resemble. |
| **9** | She continues to fall off to sleep as you take her vital signs and has trouble answering questions you ask her. |
| **10** | When she does try to answer her speech is at times incoherent and she doesn't seem to follow what you have said to her. |
| **11** | Diane does not appear to be listening to what you are asking and you need to repeat questions 3–4 times before getting an incoherent answer. |
| **12** | She tends to repeat answers from previous questions you asked. |
| **13** | Her daughter states that she does get forgetful but not like she is now. |

^a^ Each sentence in the case description is numbered for easy reference.

^b^ This case vignette was originally created and validated for geriatric nursing education research.^1^

Please evaluate this case using the Sour Seven Questionnaire^2^ below and indicate whether each of the seven observations is present (Yes/No). If you select **Yes**, please specify the sentence number(s) that informed your decision. If there are no relevant case descriptions, please select **No** and enter “NA” for the sentence number. You can use the **Comments (Optional)** column to provide more information about your reasoning or share any additional thoughts.

| Sour Seven Items | Yes | No | Sentence #, e.g., 1, 2, 11, etc. | Comments (Optional) |
| --- | --- | --- | --- | --- |
| Altered level of awareness to the environment in any way different than being normally awake. | 3 points | 0 point | 9 |  |
| Reduced attentiveness; inability to focus on you during the interaction | 4 points | 0 point | 10,11,12 |  |
| Fluctuation in awareness and attentiveness, such as drifting in and out during an interaction or through the day. | 3 points | 0 point | 10,11,12 |  |
| Disordered thinking; the response (whether verbal or action) is unrelated to the  question or request. | 3 points | 0 point | 10, 8 |  |
| Disorganized behaviour; purposeless, irrational, under-responsive or over-responsive to requests. | 2 points | 0 point | 11, 12 |  |
| Unexplained impaired eating or drinking (excluding appetite); unable to perform the actions to feed oneself. | 2 points | 0 point | 2 |  |
| Unexplained difficulty with mobility or movement. | 1 point | 0 point |  |  |
| Total Score ^a^ | 17 **Points** | | | |

^a^ Total score: >=4 suggests “possible delirium: evaluate potential medical causes, meds/substances”; 9>= suggests “delirium: immediate medical evaluation required”

**Case 4: Brian**

| **No.^a^** | **Case vignette description^b^** |
| --- | --- |
| **1** | **Brian** is an 82 year old retired store manager who is admitted to the hospital from a residential aged care facility for a total hip replacement for osteoarthritis. |
| **2** | His family says that his memory has been getting worse over the past year. |
| **3** | He now gets lost within the facility he lives in, has become neglectful of his appearance and has word finding difficulties. |
| **4** | His past history includes well controlled type 2 diabetes and osteoporosis. |
| **5** | He wears glasses and a hearing aid. |
| **6** | His regular medications include, metformin 500 mg twice a day and alendronate (Fosamax) weekly for his osteoporosis. |
| **7** | After his uneventful surgery he had some slight confusion and memory problems which were similar to his admission observations. |
| **8** | On the evening of the second post surgical day he had a sudden episode of increasing confusion associated with agitation and calling out to unknown people. |
| **9** | He pulled out his intravenous therapy and when you approached him and said his name he turned around quickly and had a fearful expression on his face. |
| **10** | He was noted to be disorientated to time, person and place, and when talking with him he had difficulty keeping track of what had been said. |
| **11** | Additionally, he did not respond appropriately to questions you asked. |
| **12** | This behavior was different to what he displayed in the nursing home. |

^a^ Each sentence in the case description is numbered for easy reference.

^b^ This case vignette was originally created and validated for geriatric nursing education research.^1^

Please evaluate this case using the Sour Seven Questionnaire^2^ below and indicate whether each of the seven observations is present (Yes/No). If you select **Yes**, please specify the sentence number(s) that informed your decision. If there are no relevant case descriptions, please select **No** and enter “NA” for the sentence number. You can use the **Comments (Optional)** column to provide more information about your reasoning or share any additional thoughts.

| Sour Seven Items | Yes | No | Sentence #, e.g., 1, 2, 11, etc. | Comments (Optional) |
| --- | --- | --- | --- | --- |
| Altered level of awareness to the environment in any way different than being normally awake. | 3 points | 0 point |  | Maybe #9 but unclear |
| Reduced attentiveness; inability to focus on you during the interaction | 4 points | 0 point | 10 |  |
| Fluctuation in awareness and attentiveness, such as drifting in and out during an interaction or through the day. | 3 points | 0 point | 10, 8, 9 |  |
| Disordered thinking; the response (whether verbal or action) is unrelated to the  question or request. | 3 points | 0 point | 9, 11 |  |
| Disorganized behaviour; purposeless, irrational, under-responsive or over-responsive to requests. | 2 points | 0 point | 9, 11 |  |
| Unexplained impaired eating or drinking (excluding appetite); unable to perform the actions to feed oneself. | 2 points | 0 point |  |  |
| Unexplained difficulty with mobility or movement. | 1 point | 0 point |  |  |
| Total Score ^a^ | 12 **Points** | | | |

^a^ Total score: >=4 suggests “possible delirium: evaluate potential medical causes, meds/substances”; 9>= suggests “delirium: immediate medical evaluation required”

**Case 5: Evan**

| **No.^a^** | **Case vignette description^b^** |
| --- | --- |
| **1** | **Evan** is a 66 year old recently retired bus driver who is admitted preoperatively to hospital for elective knee replacement surgery. |
| **2** | Following discussion with his family and himself the surgeon referred Evan to the aged care team for assessment of his ongoing memory problems. |
| **3** | Evan had recently retired from his employment because over the last twelve months he was finding it harder to remember his normal bus routes and even what hours he was supposed to work. |
| **4** | He now gets frustrated attempting to do crosswords puzzles when last year he had no difficulty completing them. |
| **5** | His family stated that he was becoming more disorganized and is newly neglectful of his appearance. |
| **6** | Additionally he has word finding difficulties and problems remembering recent events all of which had been occurring slowly. |
| **7** | He has no other significant medical history and his laboratory and physical examinations all appear normal for his age, weight and gender. |

^a^ Each sentence in the case description is numbered for easy reference.

^b^ This case vignette was originally created and validated for geriatric nursing education research.^1^

Please evaluate this case using the Sour Seven Questionnaire^2^ below and indicate whether each of the seven observations is present (Yes/No). If you select **Yes**, please specify the sentence number(s) that informed your decision. If there are no relevant case descriptions, please select **No** and enter “NA” for the sentence number. You can use the **Comments (Optional)** column to provide more information about your reasoning or share any additional thoughts.

| Sour Seven Items | Yes | No | Sentence #, e.g., 1, 2, 11, etc. | Comments (Optional) |
| --- | --- | --- | --- | --- |
| Altered level of awareness to the environment in any way different than being normally awake. | 3 points | 0 point |  |  |
| Reduced attentiveness; inability to focus on you during the interaction | 4 points | 0 point |  |  |
| Fluctuation in awareness and attentiveness, such as drifting in and out during an interaction or through the day. | 3 points | 0 point |  |  |
| Disordered thinking; the response (whether verbal or action) is unrelated to the  question or request. | 3 points | 0 point |  |  |
| Disorganized behaviour; purposeless, irrational, under-responsive or over-responsive to requests. | 2 points | 0 point |  |  |
| Unexplained impaired eating or drinking (excluding appetite); unable to perform the actions to feed oneself. | 2 points | 0 point |  |  |
| Unexplained difficulty with mobility or movement. | 1 point | 0 point |  |  |
| Total Score ^a^ | 0 **Points** | | | |

^a^ Total score: >=4 suggests “possible delirium: evaluate potential medical causes, meds/substances”; 9>= suggests “delirium: immediate medical evaluation required”

**References**

1. McCrow J, Beattie E, Sullivan K, Fick DM. Development and review of vignettes representing older people with cognitive impairment. *Geriatr Nur (Lond)*. 2013;34(2):128-137. doi:10.1016/j.gerinurse.2012.12.012

2. Shulman RW, Kalra S, Jiang JZ. Validation of the Sour Seven Questionnaire for screening delirium in hospitalized seniors by informal caregivers and untrained nurses. *BMC Geriatr*. 2016;16. doi:10.1186/s12877-016-0217-2
