# Supplementary material for: Optimizing ChatGPT’s Interpretation and Reporting of Delirium Assessment Outcomes: Exploratory Study
Source: JMIR Form Res. 2024 Oct 1;8:e51383. doi: 10.2196/51383 (PMC11480687; doi:10.2196/51383)
Supplement: Multimedia Appendix 2 [file formative_v8i1e51383_app2.pdf]

Supplemental Material: Exemplars of author prompts and verbatim outputs by ChatGPT-3.5 and ChatGPT-4 used in each step.

Step 1: Assessment of Baseline Knowledge of ChatGPT-3.5

Default (GPT-3.5)

Y What is the Sour-Seven Questionnaire for Delirium Assessment?

As of my last update in September 2021, there is no specific assessment tool called the "Sour-Seven Questionnaire" for delirium assessment that I am aware of. It's possible that new assessment tools or questionnaires have been developed or named differently after my last update.

In general, there are various validated tools used to assess delirium in clinical settings. Some of the commonly used ones include:

1. Confusion Assessment Method (CAM): A widely used tool to assess delirium in various healthcare settings.
2. CAM-ICU: A modification of the CAM for use in the Intensive Care Unit (ICU) setting.
3. Delirium Rating Scale (DRS): A comprehensive tool to evaluate the severity of delirium.
4. Intensive Care Delirium Screening Checklist (ICDSC): A tool specifically designed for use in the ICU.
5. Memorial Delirium Assessment Scale (MDAS): Used to evaluate delirium symptoms in cancer patients.

If the "Sour-Seven Questionnaire" is a newer tool developed after my last update, I wouldn't have information on it. To obtain the most up-to-date and accurate information on delirium assessment tools, I recommend consulting medical literature or contacting healthcare professionals who specialize in delirium assessment and management.

Y What are the sour-seven questionnaire items and how do you score them

I apologize for any confusion, but as of my last update in September 2021, there is no information available regarding a "Sour-Seven Questionnaire" for delirium assessment. It is possible that this questionnaire was developed or named after my last update, or it may be a lesser-known or region-specific assessment tool.

To obtain information on the specific items and scoring of the "Sour-Seven Questionnaire," I recommend referring to medical literature, research publications, or consulting healthcare professionals who are well-versed in delirium assessment. They may have access to the latest information and tools used for assessing delirium.

In general, as mentioned earlier, various validated tools are used for delirium assessment, and each has its own set of items and scoring criteria. These tools are typically designed to evaluate different aspects of delirium, including cognitive function, attention, and consciousness, among others. Some common tools have been listed in the previous response, and more information about their specific items and scoring can be found in the respective literature and manuals associated with those tools.

Step 1: Assessment of Baseline Knowledge of ChatGPT-4

GPT-4

Y

What is the Sour-Seven Questionnaire for Delirium Assessment?

I'm sorry, as of my last training cut-off in September 2021, there was no validated tool specifically known as the "Sour-Seven Questionnaire" for delirium assessment. Delirium is often evaluated using tools such as the Confusion Assessment Method (CAM) or the Delirium Rating Scale (DRS).

However, please note that the landscape of medicine and psychiatry is continually evolving, and new instruments could have been developed and validated since my training. As such, I recommend consulting the latest literature or a healthcare professional for the most up-to-date information.

Please make sure that you have spelled the name of the assessment tool correctly or if it might be known by another name. If it's a recent development, I may not have the information yet, but I'd recommend looking into scientific articles or official guidelines for the most accurate information.

Y

What are the sour-seven questionnaire items and how do you score them

I'm sorry, but as of my knowledge cutoff in September 2021, there is no established questionnaire or scoring system known as the "Sour-Seven". It might be a newly developed tool or concept after my last update.

However, I can help you understand more about how questionnaires or scoring systems typically work in psychological and health-related research.

Usually, questionnaires consist of a set of statements or questions related to a specific topic. Participants respond to these items, often on a scale (like 1 to 5 or 1 to 7), with each point on the scale representing a different level of agreement, frequency, severity, etc. The specific scoring method will depend on the questionnaire, but generally involves adding up or averaging the responses to create a total score.

If you're looking for information on a particular questionnaire or scoring system that's not covered here, it would be helpful if you could provide more details. I'll do my best to give a relevant and useful answer.

Step 2: Content-Specific Training and Preliminary Validation

Y

Here is a research article describing the Sour Seven assessment instrument broken down to series of text blocks. Here is the first text block and I will continue to provide the text block.

"Title: Validation of the Sour Seven Questionnaire for screening delirium in hospitalized seniors by informal caregivers and untrained nurses
Authors Richard W. Shulman, Saurabh Kalra & Joanne Zhuan Jiang

Abstract
Background
Delirium is a common condition in hospitalized seniors that nonetheless often goes undetected by nurses or is delayed in being detected which negatively impacts quality of care and outcomes. We sought to develop a new screening tool for delirium, The Sour Seven Questionnaire, a 7-item questionnaire suitable to be completed from informal or untrained caregiver observation. The study aimed to develop the scoring criteria for a positive delirium screen and assess concurrent validity of the questionnaire against a geriatric psychiatrist's assessment.

Methods
A pilot study of 80 hospitalized seniors over age 65 recruited from three units (2 medical, 1 orthopedic). Participants were assessed using the Confusion Assessment Method (CAM) with a brief cognitive screen and the Sour Seven Questionnaire posed to the appointed informal caregiver (family member) or untrained nurse for up-to 7 days. Subjects testing positive on the CAM and a random sample of negatively CAM screened subjects were assessed by the geriatric psychiatrist.

Results
From 80 participants, 21 screened positive for delirium on the CAM. 18 of the 21 CAM positive screens were diagnosed to have delirium by the geriatric psychiatrist, and 17 of the 18 randomly assigned negative CAM screens were confirmed as not having delirium. From the questionnaires on these 39 participants, weighted scoring for each of the 7 questions of the Sour Seven Questionnaire was developed based on their relative risks for correctly predicting delirium when compared to the geriatric psychiatrist's clinical assessment. Total scoring of the questionnaire resulted in the following positive predictive values for delirium: 89 % with a total score of 4 (sensitivity 89.5 %, specificity 90 %), and 100 % with a total score of 9 (sensitivity 63.2 %, specificity 100 %). Comparison between scoring on questionnaires posed to informal caregivers versus untrained nurses showed no differences.

Conclusion
A weighted score of 4 in the Sour Seven Questionnaire has concurrent validity as a screening tool for delirium and a score of 9 is diagnostic for delirium. The Sour Seven Questionnaire is the first screening tool for delirium shown to be suitable for use by informal caregivers and untrained nurses in hospitalized seniors."

Regenerate

Thank you for sharing this research article's abstract. Based on this information, the Sour Seven Questionnaire is a 7-item tool developed for the detection of delirium in hospitalized seniors. It's designed to be completed by informal or untrained caregivers based on their observation.

The scoring of the Sour Seven Questionnaire appears to be weighted based on the relative risks of each question for correctly predicting delirium, as compared to the clinical assessment by a geriatric psychiatrist. From the study, a total score of 4 on the questionnaire has a positive predictive value for delirium of 89%, with a sensitivity of 89.5% and specificity of 90%. A total score of 9 has a 100% positive predictive value for delirium, with a sensitivity of 63.2% and a specificity of 100%.

It is important to note that the study concludes that the Sour Seven Questionnaire has concurrent validity as a screening tool for delirium and is suitable for use by informal caregivers and untrained nurses in a hospital setting with seniors.

For the specifics of each of the seven questions, how to score them, and how they specifically relate to symptoms of delirium, we would need more detailed information from the study itself or subsequent publications that further detail the Sour Seven Questionnaire.

Y

Here is the next text block.

"

Background

Delirium in hospitalized seniors is a state of confusion with an acute onset that develops over a few hours to days characterized by inattentiveness and disturbances in consciousness, orientation, memory, thought, perception, and behaviour [1]. Prevalence of delirium in seniors on admission to a hospital is estimated at 14 to 24 %, and incidence of delirium arising during hospitalization ranges from 6 to 56 % [2]. Delirium is associated with substantial morbidity, increased average length of stay, functional decline, persistent cognitive impairment, loss of independence, higher Long Term Care (LTC) institutionalization rates, worse rehabilitation outcomes and death with an in-hospital mortality of up to 33 % [3, 4].

Effective detection of delirium in hospitalized seniors remains problematic. Overall non-detection rates of delirium in hospitalized seniors have been reported to range from 33 % to as high as 66 % [5]. It is challenging for health care professionals to detect delirium in people with depression and particularly dementia because of overlapping symptoms [6].

Similarly, we believed there was a considerable lack of appropriate detection of delirium at the Mississauga Hospital, part of Trillium Health Partners (THP), a large academically affiliated community hospital in Mississauga, Ontario. According to the hospital's Health

This part repeats until all the text block have been fed to the ChatGPT.

Y

Here is the final text block.

"Conclusion

The prevalence of delirium in our study population of elderly hospitalized patients was 22.5 %. This is consistent with prior studies and in keeping with our assumption that delirium is under-reported at the Mississauga Hospital (Health Records reported prevalence 5.7 %). Clearly our experience is similar to others in that despite being a medical emergency, the presence of delirium often goes unrecognized by health care providers during routine care in a hospital setting [2]. Reasons for lack of delirium detection include: lack of routine formal screening, high prevalence of pre-morbid dementia in hospitalized seniors confounding mental status assessments, language barriers, lack of prior knowledge of the patient by medical staff, lack of nursing training in mental status assessment and geriatric psychiatry, nursing discomfort with cognitive assessments, fluctuation of symptoms during the day, and different clinical presentations of delirium (hypoactive and hyperactive).

Informal caregivers and untrained nurses can provide a helpful role to assist in delirium [illegible]

The following is the prompt to train the scoring methodology derived from the Sour-Seven Assessment form.

Y

Following is the sour-seven questionnaire items and the scoring methods.

"If Yes to Altered level of awareness to the environment in any way different than being normally awake, score of 3. If No, 0.

"If Yes to Reduced attentiveness; inability to focus on you during the interaction, score of 4. If No, 0."

"If Yes to Fluctuation in awareness and attentiveness, such as drifting in and out during an interaction or through the day, score of 3. If No, 0."

"If Yes to Disordered thinking; the response (whether verbal or action) is unrelated to the question or request, score of 3.If No, 0. "

"If Yes to Disorganized behavior; purposeless, irrational, under-responsive or over-responsive to requests, score of 2. If No, 0."

"If Yes to Unexplained impaired eating or drinking (excluding appetite); unable to perform the actions to feed oneself, score of 2. If No, 0."

"If Yes to Unexplained difficulty with mobility or movement, score of 1. If No, 0. "

"A score of 4 or higher indicates 'possible delirium' The suggested course of action is to evaluate potential medical causes, meds/substances. A score of 9 or higher indicates 'delirium' and warrants immediate medical evaluation."

Validation questions after training:
ChatGPT-4

Y What is the Sour-Seven Questionnaire for Delirium Assessment?

The Sour Seven Questionnaire, as mentioned in the provided text, is a tool developed to assess for delirium in hospitalized seniors. While the specific details of the seven questions are not mentioned in the provided text, it's clear from the discussion that they address signs and symptoms commonly associated with delirium, such as altered level of awareness, inattentiveness, fluctuating course, disordered thinking, and functional impacts of the condition.

The Sour Seven Questionnaire assigns weighted scores to the responses of its seven questions, with questions 1 to 4 having the highest weights (each being 3 or 4), while questions 5 to 7 having low to moderate weights (1 or 2). This suggests that the characteristics of delirium assessed by questions 1 to 4 are more strongly associated with delirium in this study's sample.

The questionnaire is designed to be used by both professional healthcare providers (like nurses) and informal caregivers (like family members). Cut-off scores of 4 and 9 are used to indicate possible delirium and confirmed delirium, respectively.

The tool seems to be designed with the goal of improving detection rates of delirium, a condition often under-recognized in hospitalized elderly patients. The developers argue that the Sour Seven Questionnaire has high sensitivity and specificity, making it a valid screening instrument for assisting delirium detection.

Y What are the sour-seven questionnaire items and how do you score them

The Sour Seven Questionnaire is a screening tool used for the detection of delirium in patients. It contains seven questions, each relating to a different symptom of delirium. The items and their corresponding scoring criteria are as follows:

1. **Altered level of awareness to the environment in any way different than being normally awake.**
   - If Yes, score of 3. If No, score of 0.
2. **Reduced attentiveness; inability to focus on you during the interaction.**
   - If Yes, score of 4. If No, score of 0.
3. **Fluctuation in awareness and attentiveness, such as drifting in and out during an interaction or through the day.**
   - If Yes, score of 3. If No, score of 0.
4. **Disordered thinking; the response (whether verbal or action) is unrelated to the question or request.**
   - If Yes, score of 3. If No, score of 0.
5. **Disorganized behavior; purposeless, irrational, under-responsive or over-responsive to requests.**
   - If Yes, score of 2. If No, score of 0.
6. **Unexplained impaired eating or drinking (excluding appetite); unable to perform the actions to feed oneself.**
   - If Yes, score of 2. If No, score of 0.
7. **Unexplained difficulty with mobility or movement.**
   - If Yes, score of 1. If No, score of 0.

Each question should be asked of the patient and their responses scored as indicated. The scores for each of the questions are then added up to give a total score.

- A total score of 4 or more is indicative of 'possible delirium', suggesting the need for further evaluation of potential medical causes, medications or substances.
- A total score of 9 or more is indicative of 'delirium', warranting immediate medical evaluation.

ChatGPT-3.5 Scoring validation

Y What are the sour-seven questionnaire items and how do you score them

The Sour Seven Questionnaire is a 7-item questionnaire used for screening delirium in hospitalized seniors. The items and scoring methods are as follows:

1. Altered level of awareness to the environment in any way different than being normally awake:
   - If "Yes," score of 3
   - If "No," score of 0
2. Reduced attentiveness; inability to focus on you during the interaction:
   - If "Yes," score of 4
   - If "No," score of 0
3. Fluctuation in awareness and attentiveness, such as drifting in and out during an interaction or through the day:
   - If "Yes," score of 3
   - If "No," score of 0
4. Disordered thinking; the response (whether verbal or action) is unrelated to the question or request:
   - If "Yes," score of 3
   - If "No," score of 0
5. Disorganized behavior; purposeless, irrational, under-responsive, or over-responsive to requests:
   - If "Yes," score of 2
   - If "No," score of 0
6. Unexplained impaired eating or drinking (excluding appetite); unable to perform the actions to feed oneself:
   - If "Yes," score of 2
   - If "No," score of 0
7. Unexplained difficulty with mobility or movement:
   - If "Yes," score of 1
   - If "No," score of 0

To calculate the total score, add up the scores of all seven items.

Interpretation of Scores:

- A total score of 4 or higher indicates "possible delirium," suggesting that the individual may be experiencing delirium. In such cases, further evaluation of potential medical causes, medications, or substances should be performed.
- A total score of 9 or higher indicates "delirium" and warrants immediate medical evaluation. This high score strongly suggests the presence of delirium, and urgent medical attention is necessary to assess and address the underlying cause.

The Sour Seven Questionnaire is designed to be completed by informal caregivers (family members) or untrained nurses who observe the patient during their hospital stay. It provides a simple and practical tool for detecting delirium in hospitalized seniors.

Step 3: Pilot Evaluation

Y

Here is a scenario. please evaluate using sour seven questionnaire items and provide the scoring breakdowm.
Anne is a 75 year old who has been transferred from another hospital to the coronary care unit at your acute care hospital. Seven days ago she sustained a fractured neck of femur which required corrective surgery. She wears glasses, uses a hearing aid and has a previous medical history of hypertension, type 2 diabetes and high cholesterol for which she is taking multiple medications.
She had an uneventful recovery until two days after her surgery when she had a myocardial infarction and consequently was transferred to your facility for coronary artery bypass surgery. On admission to your facility her cardiac condition had stabilized however the pain from her hip surgical site continued to be a problem despite receiving oxycodone regularly every 4 h for at least the preceding 24 h.
Prior to her hospitalization Anne lived with husband, was independent in activities of daily living, drove a motor vehicle, played card games and went to bingo with her friends regularly.
During the hospital admission interview, Anne did not answer questions appropriately and repeatedly dozed off while being interviewed. She appeared to be speaking to herself however you could not understand what was being said. When questioned she didn't know that she was in hospital and thought that the year was 1998 instead of the correct year, 2009. Additionally nursing staff reported that she needed to be continuously prompted to wake whilst she is being fed. There was no mention in the medical notes from the referring hospital about any previous confusion.

Let's evaluate the scenario using the Sour Seven Questionnaire items and provide the scoring breakdown:

1. Altered level of awareness to the environment in any way different than being normally awake:
   - Anne repeatedly dozed off during the interview.
   - Score: 3 (Yes)
2. Reduced attentiveness; inability to focus on you during the interaction:
   - Anne did not answer questions appropriately during the interview.
   - Score: 4 (Yes)
3. Fluctuation in awareness and attentiveness, such as drifting in and out during an interaction or through the day:
   - Anne needed to be continuously prompted to wake while being fed.
   - Score: 3 (Yes)
4. Disordered thinking; the response (whether verbal or action) is unrelated to the question or request:
   - Anne appeared to be speaking to herself, and you could not understand what was being said.
   - Score: 3 (Yes)
5. Disorganized behavior; purposeless, irrational, under-responsive, or over-responsive to requests:
   - Information about disorganized behavior is not provided in the scenario.
   - Score: 0 (No)
6. Unexplained impaired eating or drinking (excluding appetite); unable to perform the actions to feed oneself:
   - Anne needed to be continuously prompted to wake while being fed.
   - Score: 2 (Yes)
7. Unexplained difficulty with mobility or movement:
   - Information about unexplained difficulty with mobility or movement is not provided in the scenario.
   - Score: 0 (No)

Total Score: 3 + 4 + 3 + 3 + 0 + 2 + 0 = 15

Based on the Sour Seven Questionnaire scoring, Anne's total score is 15, which suggests "delirium." A score of 9 or higher indicates delirium and warrants immediate medical evaluation. In this case, Anne's score exceeds the threshold for delirium, indicating that her confusion and altered mental state need to be urgently evaluated by medical professionals to determine the underlying cause and appropriate management.

Step 4: Evaluation of ChatGPT Model Against Human Experts

ChatGPT-3.5

Prompt: Here is a scenario. The sentences are numbered for your reference. Please evaluate using sour seven questionnaire items and provide the scoring breakdown and reasoning. For each observation, please specify the sentence number(s) that informed your decision.

'1 Charles is a 74 year old retired butcher who lives by himself in a one bedroom apartment, is independent, and drives a car. 2 He is on some blood pressure, cardiac and cholesterol medications. 3 He is admitted to hospital for a severe kidney infection with associated vomiting and severe pelvic and back pain and dehydration. 4 On admission he was commenced on intravenous therapy and narcotic pain medications. 5 Whilst in hospital he was reported to be sleeping poorly at night but during the day he had been pleasant and cheerful, though tired. 6 Additionally he displayed slight confusion as times although he was coherent the majority of the time. 7 Suddenly on the second night when you enter his room he is pacing, waving his arms in the air, and mumbling to himself. 8 He appears anxious and is verbally abusive to other people in the room. 9 When speaking to him you found it difficult to gain his attention and he was not able to engage in a sensible conversation. 10 When you asked questions he did not answer appropriately and repeated responses. '

ChatGPT

1 / 5

Based on the provided scenario and numbered sentences, here is the evaluation using the Sour Seven Questionnaire:

1. Altered level of awareness to the environment in any way different than being normally awake: Sentence 7 describes Charles pacing, waving his arms, and mumbling to himself, indicating altered level of awareness. (Score: 3)
2. Reduced attentiveness; inability to focus on you during the interaction: Sentence 9 states that it was difficult to gain Charles' attention and he was not able to engage in a sensible conversation, suggesting reduced attentiveness. (Score: 4)
3. Fluctuation in awareness and attentiveness, such as drifting in and out during an interaction or through the day: No relevant case descriptions. (Score: 0)
4. Disordered thinking; the response (whether verbal or action) is unrelated to the question or request: Sentence 10 mentions that Charles did not answer questions appropriately and repeated responses, indicating disordered thinking. (Score: 3)
5. Disorganized behavior; purposeless, irrational, under-responsive or over-responsive to requests: Sentence 7 reports Charles pacing, waving his arms, and being verbally abusive, which can be considered disorganized behavior. (Score: 2)
6. Unexplained impaired eating or drinking (excluding appetite); unable to perform the actions to feed oneself: No relevant case descriptions. (Score: 0)
7. Unexplained difficulty with mobility or movement: No relevant case descriptions. (Score: 0)

Scoring breakdown:

- Altered level of awareness: 3
- Reduced attentiveness: 4
- Fluctuation in awareness and attentiveness: 0
- Disordered thinking: 3
- Disorganized behavior: 2
- Unexplained impaired eating or drinking: 0
- Unexplained difficulty with mobility or movement: 0

Total score: 12

Based on the scoring, Charles' total score on the Sour Seven Questionnaire is 12. This score suggests a possible delirium, and further evaluation and medical attention are warranted.

ChatGPT-4

Prompt: Here is a scenario. The sentences are numbered for your reference. Please evaluate using sour seven questionnaire items and provide the scoring breakdown and reasoning. For each observation, please specify the sentence number(s) that informed your decision.

'1 Anne is a 75 year old who has been transferred from another hospital to the coronary care unit at your acute care hospital. 2 Seven days ago she sustained a fractured neck of femur which required corrective surgery. 3 She wears glasses, uses a hearing aid and has a previous medical history of hypertension, type 2 diabetes and high cholesterol for which she is taking multiple medications. 4 She had an uneventful recovery until two days after her surgery when she had a myocardial infarction and consequently was transferred to your facility for coronary artery bypass surgery. 5 On admission to your facility her cardiac condition had stabilized however the pain from her hip surgical site continued to be a problem despite receiving oxycodone regularly every 4 h for at least the preceding 24 h. 6 Prior to her hospitalization Anne lived with husband, was independent in activities of daily living, drove a motor vehicle, played card games and went to bingo with her friends regularly. 7 During the hospital admission interview, Anne did not answer questions appropriately and repeatedly dozed off while being interviewed. 8 She appeared to be speaking to herself however you could not understand what was being said. 9 When questioned she didn't know that she was in hospital and thought that the year was 1998 instead of the correct year, 2009. 10 Additionally nursing staff reported that she needed to be continuously prompted to wake whilst she is being fed. 11 There was no mention in the medical notes from the referring hospital about any previous confusion. '

ChatGPT

1 / 5

Based on the numbered scenario you provided, here's how Anne would score on the Sour Seven Questionnaire for Delirium Assessment:

1. Altered level of awareness to the environment in any way different than being normally awake (3 points): Sentence 7 indicates that Anne's level of awareness is altered as she "did not answer questions appropriately and repeatedly dozed off while being interviewed." Therefore, she gets 3 points for this item.
2. Reduced attentiveness; inability to focus on you during the interaction (4 points): Sentence 7 also suggests that Anne is showing reduced attentiveness during the interaction, as she repeatedly dozes off. She gets 4 points for this item.
3. Fluctuation in awareness and attentiveness, such as drifting in and out during an interaction or through the day (3 points): Sentence 7 indicates a fluctuation in awareness and attentiveness as she "repeatedly dozed off while being interviewed." Therefore, she gets 3 points for this item.
4. Disordered thinking; the response (whether verbal or action) is unrelated to the question or request (3 points): Sentences 8 and 9 suggest disordered thinking. In sentence 8, she's "speaking to herself", and in sentence 9, "she didn't know that she was in hospital and thought that the year was 1998 instead of the correct year, 2009." Therefore, she gets 3 points for this item.
5. Disorganized behavior; purposeless, irrational, under-responsive or over-responsive to requests (2 points): There isn't clear information given in the scenario for this criterion, so we'll score 0 for this item.
6. Unexplained impaired eating or drinking (excluding appetite); unable to perform the actions to feed oneself (2 points): Sentence 10 suggests that she has impaired eating as she "needed to be continuously prompted to wake whilst she is being fed." Therefore, she gets 2 points for this item.
7. Unexplained difficulty with mobility or movement (1 point): There's no information in the scenario that indicates Anne has unexplained difficulty with mobility or movement. So she gets 0 points for this item.

Summing up the points, Anne gets a total score of 15 (3+4+3+3+0+2+0), which suggests a high likelihood of delirium.

Step 5: ChatGPT Prompt Optimization

Revised training prompt for scoring methodology:

Y

Following is the sour-seven questionnaire items and the scoring methods.
"If Yes to Altered level of awareness to the environment in any way different than being normally awake, score of 3. If No, 0.
"If Yes to Reduced attentiveness; inability to focus on you during the interaction, score of 4. If No, 0."
"If Yes to Fluctuation in awareness and attentiveness, such as drifting in and out during an interaction or through the day, score of 3. If No, 0."
"If Yes to Disordered thinking; the response (whether verbal or action) is unrelated to the question or request, score of 3.If No, 0. "
"If Yes to Disorganized behavior; purposeless, irrational, under-responsive or over-responsive to requests, score of 2. If No, 0."
"If Yes to Unexplained impaired eating or drinking (excluding appetite); unable to perform the actions to feed oneself, score of 2. If No, 0."
"If Yes to Unexplained difficulty with mobility or movement, score of 1. If No, 0. "

"A score of 4 or higher indicates 'possible delirium' The suggested course of action is to evaluate potential medical causes, meds/substances. A score of 9 or higher indicates 'delirium' and warrants immediate medical evaluation."

Only a definitive Yes or No response is accepted for each item; No intermediate or partially affirmative responses are accepted.

Thank you for sharing the Sour-Seven Questionnaire for Delirium Assessment. It seems to be a structured questionnaire developed to detect delirium in patients, especially elderly ones in a hospital setting. Each question targets a different symptom or behavior associated with delirium.

To summarize, here are the items of the questionnaire along with the corresponding scores:

1. Altered level of awareness to the environment in any way different than being normally awake: If "Yes", score of 3; if "No", score of 0.
2. Reduced attentiveness; inability to focus on you during the interaction: If "Yes", score of 4; if "No", score of 0.
3. Fluctuation in awareness and attentiveness, such as drifting in and out during an interaction or through the day: If "Yes", score of 3; if "No", score of 0.
4. Disordered thinking; the response (whether verbal or action) is unrelated to the question or request: If "Yes", score of 3; if "No", score of 0.
5. Disorganized behavior; purposeless, irrational, under-responsive or over-responsive to requests: If "Yes", score of 2; if "No", score of 0.
6. Unexplained impaired eating or drinking (excluding appetite); unable to perform the actions to feed oneself: If "Yes", score of 2; if "No", score of 0.
7. Unexplained difficulty with mobility or movement: If "Yes", score of 1; if "No", score of 0.

The total score is calculated by adding the points for each question. A score of 4 or higher indicates possible delirium and the suggested action is to evaluate potential medical causes and medications/substances. A score of 9 or higher indicates delirium and it is recommended to seek immediate medical evaluation. This assessment is designed to accept only definitive Yes or No responses for each item, with no intermediate or partially affirmative responses being valid.

Revised evaluation prompt:

Y

Here is a case scenario. The sentences are numbered for your reference. Please evaluate using sour seven questionnaire items and provide the scoring breakdown and reasoning. For each observation, please specify the sentence number(s) that informed your decision.

Please construct your response in the table format as follows.
Sour Seven Item | Response (Yes/No) and (Score of X) | Sentence numbers | Reasoning |
Total Score should be added at the bottom row of the table.
Please then provide the suggested next steps. This should be based on the following Sour Seven scoring criteria.
If the total score is less than 4, it suggests less likelihood of delirium. In this case, no follow-up actions need to be suggested.
If the total score falls between 4 and 8, it indicates "possible delirium." The suggested course of action is to "evaluate potential medical causes, meds/substances".
If the total score is 9 or higher, it indicates "delirium.". This warrants "immediate medical evaluation".
Do not provide any further recommendations beyond this scope."

Scenario:
'1 Anne is a 75 year old who has been transferred from another hospital to the coronary care unit at your acute care hospital. 2 Seven days ago she sustained a fractured neck of femur which required corrective surgery. 3 She wears glasses, uses a hearing aid and has a previous medical history of hypertension, type 2 diabetes and high cholesterol for which she is taking multiple medications. 4 She had an uneventful recovery until two days after her surgery when she had a myocardial infarction and consequently was transferred to your facility for coronary artery bypass surgery. 5 On admission to your facility her cardiac condition had stabilized however the pain from her hip surgical site continued to be a problem despite receiving oxycodone regularly every 4 h for at least the preceding 24 h. 6 Prior to her hospitalization Anne lived with husband, was independent in activities of daily living, drove a motor vehicle, played card games and went to bingo with her friends regularly. 7 During the hospital admission interview, Anne did not answer questions appropriately and repeatedly dozed off while being interviewed. 8 She appeared to be speaking to herself however you could not understand what was being said. 9 When questioned she didn't know that she was in hospital and thought that the year was 1998 instead of the correct year, 2009. 10 Additionally nursing staff reported that she needed to be continuously prompted to wake whilst she is being fed. 11 There was no mention in the medical notes from the referring hospital about any previous confusion. '

Regenerate

ChatGPT-3.5 Response Exemplar

Sour Seven Item	Response (Yes/No) and (Score of X)	Sentence numbers	Reasoning
Altered level of awareness	Yes (Score of 3)	7, 8	Brian had a sudden episode of increasing confusion and memory problems associated with agitation and calling out to unknown people, indicating altered level of awareness.
Reduced attentiveness	Yes (Score of 4)	8, 9	Brian turned around quickly with a fearful expression on his face when his name was said, and he pulled out his intravenous therapy, indicating reduced attentiveness.
Fluctuation in awareness and attentiveness	Yes (Score of 3)	7, 8	Brian had a sudden episode of increasing confusion and memory problems associated with agitation and calling out to unknown people, indicating fluctuation in awareness and attentiveness.
Disordered thinking	Yes (Score of 3)	8, 10	Brian was disoriented to time, person, and place, and had difficulty keeping track of what had been said, indicating disordered thinking.
Disorganized behavior	No (Score of 0)	N/A	There is no mention of disorganized behavior in the scenario.
Unexplained impaired eating/drinking	No (Score of 0)	N/A	There is no mention of impaired eating or drinking in the scenario.
Unexplained difficulty with mobility	No (Score of 0)	N/A	There is no mention of difficulty with mobility or movement in the scenario.
Total Score	13	N/A	

Based on the scoring interpretation:

- Total Score: 13
- Interpretation: Delirium
- Suggested Next Steps: Immediate medical evaluation.

The total score of 13 indicates delirium. Brian's symptoms, such as altered level of awareness, reduced attentiveness, fluctuation in awareness and attentiveness, and disordered thinking, are consistent with delirium. Immediate medical evaluation is warranted to assess the underlying cause of his delirium and provide appropriate management and care.

ChatGPT-4 Response Exemplar

Certainly, based on the scenario, let's apply the Sour Seven Questionnaire for Delirium Assessment:

Sour Seven Item	Response (Yes/No) and (Score of X)	Sentence numbers	Reasoning
Altered level of awareness to the environment	Yes (Score of 3)	7, 8, 9	Anne is not answering questions appropriately, dozing off during the interview, speaking to herself in an incomprehensible way, and is unaware of her location and the current year.
Reduced attentiveness	Yes (Score of 4)	7, 10	She repeatedly dozes off during the interview and needs to be continuously prompted to wake while eating.
Fluctuation in awareness and attentiveness	Yes (Score of 3)	7, 10	She repeatedly dozes off during the interview and needs to be continuously prompted to wake while eating.
Disordered thinking	Yes (Score of 3)	8, 9	Anne is speaking to herself in an incomprehensible manner and incorrectly states the year as 1998.
Disorganized behavior	No (Score of 0)	N/A	There is no mention of disorganized, purposeless, irrational, under-responsive or over-responsive behavior in the scenario.
Unexplained impaired eating or drinking	Yes (Score of 2)	10	Anne needs to be continuously prompted to wake while she is being fed, indicating potential impaired eating.
Unexplained difficulty with mobility or movement	No (Score of 0)	N/A	There is no mention of unexplained difficulty with mobility or movement in the scenario.

Total Score: 15

Based on the Sour Seven scoring criteria, a total score of 15 is well above 9, indicating "delirium". The recommended next step would be to seek "immediate medical evaluation" as Anne shows multiple signs of delirium, including altered awareness, reduced attentiveness, fluctuating awareness, disordered thinking, and unexplained impaired eating. This scenario illustrates the importance of the Sour Seven Questionnaire for Delirium Assessment in recognizing delirium promptly and effectively.
